# Molecular Research of Glycolysis

**DOI:** 10.3390/ijms23095052

**Published:** 2022-05-02

**Authors:** Yu-Chan Chang, Cheorl-Ho Kim

**Affiliations:** 1 Department of Biomedical Imagine and Radiological Sciences, National Yang Ming Chiao Tung University, Taipei 11221, Taiwan; 2 Molecular and Cellular Glycobiology Unit, Department of Biological Sciences, College of Science, Sungkyukwan University, Suwon City 16419, Korea; chkimbio@skku.edu

Glycolysis represents the process of breaking down monosaccharides, which involves the energy metabolism, homeostasis, and the linkage of various physiological functions such as muscle movement, development, neurotransmission, etc. Glycolysis research and perspectives have evolved over time. Still, the field has experienced several disruptions and revolutions in recent decades, as scientists treat known theories as Bible. At present, glycolysis offers the next level of glycolytic insight and reprogramming due to the interactions between physiology, pathology, tumor progression, immunity, and gut microbiota.

Glycolytic programs and rates under environmental stress and physiological conditions will be regulated and reprogrammed. Tumor hypoxia and the tumor microenvironment are regular events associated with aberrant glycolysis [1]. As immunotherapy and the immune response become a hot research topic, tumor-associated macrophages (TAM) [2], cancer-associated fibroblasts (CAFs) [3], and other immune populations [4] that interact with cancer cells and their glycolytic events will need more attention. Glycolysis-related oncometabolites/immunometabolisms will become an important research direction, not only focused on metabolic events at local tumor foci. Interestingly, the gut microbiome’s impact on immunometabolism, gastrointestinal cancers, and the gut–brain axis is also being explored [5,6]. Identifying the metabolite profile and the intestinal microbiota profile for the current paradox will provide novel therapeutic strategies [7].

The extent of glycolysis goes beyond this, involving multiple species and a wide range of functional mediators. The initiation and turnover of glycolysis respond to changes in the intracellular environment, transduction signals, and the characteristics of differentiated cells. In recent years, differences in glycolytic enzyme activities between comprehensive species or tissues have been found, which are worth exploring [8]. As a result of these findings, the circadian clock [9], neurodegenerative disorders [10] and systemic diseases (e.g., diabetes, hypertension) [11] are inextricably linked to the partnership of glycolysis.

The studies collected in this Special Issue clearly demonstrate the impact of glycolysis on various aspects of cancer cell biology and its influence on physiology. Glycolysis is very important in cancer biology as some of the intermediates in tumor metabolism can significantly affect the local tumor region and the surrounding tumor microenvironment and support various cancer hallmarks. The general contribution of these studies is as follows: (1) the aberrant performance of glycolytic enzymes in cancers is highlighted [12], (2) biostatistical analysis suggests that it has clinical prognostic indicators [12], (3) new definitions or associations between glycolytic-related events and cancer phenotypes are offered [13], (4) the molecular mechanisms of its involvement in the development of clinical strategies development are explored [14]. In addition, our Special Issues provide some further discussion topics: (1) primary or functional cell-associated metabolic programs (e.g., fibroblasts [15]), (2) the relationship between physiology and glycolysis [16], (3) discussion on the eukaryotic glycolysis system [17].

A detailed study of its distribution, key switches, and symbolic physiology will be an essential research direction in the future. Given these aspects, the research on glycolysis will not stop, and its importance will also keep develop over time.
